# A Physical Framework for Algorithmic Entropy

**DOI:** 10.3390/e28010061

**Published:** 2026-01-04

**Authors:** Jeff Edmonds

**Affiliations:** Department of Electrical Engineering and Computer Science, York University, Toronto, ON M3J 1P3, Canada; jeff@yorku.ca

**Keywords:** Kolmogorov complexity, entropy, macrostate, microstate, Levin’s Coding Theorem, phase space, determinism, coarse-graining, Liouville’s Theorem, gravity, foundational principles

## Abstract

This paper does not aim to prove new mathematical theorems or claim a fundamental unification of physics and information, but rather to provide a new pedagogical framework for interpreting foundational results in algorithmic information theory. Our focus is on understanding the profound connection between entropy and Kolmogorov complexity. We achieve this by applying these concepts to a physical model. Our work is centered on the distinction, first articulated by Boltzmann, between observable low-complexity macrostates and unobservable high-complexity microstates. We re-examine the known relationships linking complexity and probability, as detailed in works like Li and Vitányi’s *An Introduction to Kolmogorov Complexity and Its Applications*. Our contribution is to explicitly identify the abstract complexity of a probability distribution K(ρ) with the concrete physical complexity of a macrostate K(M). Using this framework, we explore the “Not Alone” principle, which states that a high-complexity microstate must belong to a large cluster of peers sharing the same simple properties. We show how this result is a natural consequence of our physical framework, thus providing a clear intuitive model for understanding how algorithmic information imposes structural constraints on physical systems. We end by exploring concrete properties in physics, resolving a few apparent paradoxes, and revealing how these laws are the statistical consequences of simple rules.

## 1. Introduction

Boltzmann’s entropy H(X) was as important to the Industrial Age as Shannon’s is to the Information Age [1,2]. Both quantify uncertainty and the spread of information. Parallel to this, Kolmogorov complexity K(x) provides a measure of the information content of an individual object as the length of the shortest computer program required to describe it [3]. The deep interconnections between these frameworks are comprehensively detailed in landmark texts, most notably Li and Vitányi’s *An Introduction to Kolmogorov Complexity and Its Applications* [4].

The results in algorithmic information theory are often powerfully abstract and mathematically dense. This paper aims to provide a **new pedagogical interpretation** of these foundational results by explicitly applying them to the physical framework of thermodynamics. We emphasize that this implies a structural analogy to aid intuition, rather than a claim that continuous physical reality is identical to discrete algorithmic strings. Our central thesis revolves around the distinction, first articulated by Boltzmann, between observable low-complexity macrostates (like the temperature of a gas) and the unobservable high-complexity microstates (the precise configuration of all particles) that comprise them.

We must be clear about our contribution. The mathematical results we discuss—including the Levin–Chaitin bound [5,6] $K(\alpha )\leq -\log_{2}\rho (\alpha )+K(\rho )+c$ and the “Not Alone” principle (c.f. [4], (Thm. 2.1.3))—are known and foundational. The novelty of this paper lies not in proving new theorems but in synthesizing them into a single intuitive framework. Our central thesis is that the abstract complexity of a distribution K(ρ) can be identified with the concrete physical complexity of an observable macrostate K(M). Though we say that a macrostate represents a collection of properties/constraints, we mean this only for intuition. Any attempt to map these properties to probabilities ρ(α) is well beyond the scope of this paper. In this framework, we explicitly identify the macrostate *M* with the code/program that generates its probability distribution ρ. Thus, the complexity of the macrostate K(M) is effectively defined as the complexity of the distribution K(ρ).

This physical framework allows us to re-derive and re-interpret these known results, demonstrating how they naturally emerge as structural constraints on physical systems. Our main application is exploring the “Not Alone” principle: we show how a microstate’s high complexity must be balanced by the size of the cluster it inhabits, where the cluster is defined by any simple computable property.

It is important to distinguish our approach from the seminal work of Zurek [7], who also integrated algorithmic randomness with physical entropy. While Zurek focused on the thermodynamic cost of measurement and the operation of heat engines, our work aims to provide a structural framework connecting the abstract properties of Kolmogorov complexity directly to the definition of macrostates and their cluster sizes.

While our framework highlights the deep structural connections between informational entropy and thermodynamic entropy, we acknowledge the ongoing debate regarding whether they are truly identical or merely analogous. Researchers such as Elitzur [8] and Meyer [9] have argued that information (or complexity) and thermodynamic “order” are distinct characteristics that should not be conflated. Elitzur posits that thermodynamic entropy measures the dispersal of energy, which is distinct from the informational content related to structure and complexity—a crystal, for example, has low entropy but low informational complexity, whereas a living organism has low entropy but high informational complexity. Meyer reinforces this by arguing that information is a measurable physical quantity distinct from thermodynamic entropy, essential for understanding biological organization. Our work takes the perspective that treating them as unified provides powerful pedagogical insights for statistical mechanics, but we respect the distinction emphasized in these works regarding biological and functional complexity.

**Our contributions can be summarized as follows:**
  **(1)**  **A Physical Framework:** An intuitive model for pedagogical purposes that explicitly identifies the abstract complexity of a distribution K(ρ) with the concrete complexity of a physical macrostate K(M).
  **(2)**  **A New Interpretation of the Not Alone Principle:** We use this framework to show how the known “Not Alone” result (c.f. [4], (Thm. 2.1.3)) arises as a natural structural constraint linking a microstate’s high complexity to the size of the cluster defined by any of its simple properties.
  **(3)**  **A Unified View:** We demonstrate how this physical interpretation connects to other foundational results including the Sandwich Theorem (H(M)≈Exp[K(α)]) and the bounded variance of complexity in uniform distributions.
  **(4)**  **Macro- vs. Microstates in Physics:** This section explores how macro-level concepts in physics are derived from the micro-level properties of particles. With this, we are able to resolve many of the field’s apparent paradoxes. This perspective reveals that the foundational laws of thermodynamics are not arbitrary but are the necessary statistical consequences of simple rules applied to a complex world, all governed by the universal logic of information.

**The paper is organized as follows: **Section 2 defines the key terms used in the theorems and illustrates each with intuition and concrete examples. Section 3 explores illustrative examples. Section 4 states the formal theorems and provides their proofs. Section 5 explores the connection to physics in more detail. Section 6 concludes. I end by acknowledging Paul Vitányi, Ming Li, and my AI collaborators.

## 2. Intuition About the Definitions and Results

In this section, we lay out the key concepts and intuitions that underlie the results of this paper, supported by concrete examples. Our goal is to offer an accessible and computationally grounded explanation of the connection between entropy and Kolmogorov complexity. Central to our perspective is the distinction between observable low-complexity macrostates and unobservable high-complexity microstates. We do not know to what extent this intuition was part of Levin’s original motivation for his Coding Theorem; however, we believe that the following explanation is an important contribution of this paper.

**The Physical World:** Boltzmann defined the macro- and microstates of a physical system like a gas [1].
**Microstate *α*** is a finite binary string encoding a physical system’s complete unobservable high-complexity configuration
  (e.g., it could encode the positions, velocities, and masses of all ≈$10^{27}$ particles).
**Properties *P*(*α*) = *p*:** Let *P* be any computable property of a microstate. For a given α, let p=P(α) be the value of that property. The set $S_{p}=\left\{\alpha^{\prime }\in S_{M}:P\left(\alpha^{\prime }\right)=p\right\}$ is the cluster of all microstates sharing that property value (e.g., the temperature of the gas).
**Macrostate *M*** represents the observable low-complexity collection of properties of the physical system (e.g., the temperature and pressure).
**Probability *ρ*(*α*)** denotes the probability Pr(α|M) of the system being in microstate α given that we know that it is in macrostate M (e.g., in a system influenced by gravity, microstates representing molecules higher up have lower probabilities ρ(α)).
***S_M_*** is the set of microstates consistent with this macrostate, namely $S_{M}=\left\{\alpha :\rho (\alpha )>0\right\}$.
**Program** $M_{M}$ for the macrostate is assumed to operate in two modes:
  - An approximation mode $M_{M}\left(\alpha ,\epsilon \right)$, which outputs an ϵ-approximation of the real-valued probability ρ(α).
  - A decision mode $M_{M}\left(\alpha \right)$, which returns {0,1} to decide membership in $S_{M}$. (It might be undecidable even with $M_{M}\left(\alpha ,\epsilon \right)$ to know whether ρ(α)> 0 because this probability might be arbitrarily small.)
  As noted above, we explicitly identify the macrostate M with the code/program that generates its probability distribution ρ.
**Entropy** H(M)**:** Boltzmann’s thermodynamics-motivated entropy [1] is defined to be the logarithm of the number of microstates consistent with the observed macrostate, namely (Physicists often use $H(M)\overset{\mathrm{def}}{=}k\ln\left|S_{M}\right|$, where k is Boltzmann’s constant. We use base-2 logarithms measuring entropy in bits, in line with Shannon and Kolmogorov).
  (1) $$\begin{array}{c} H(M)\overset{\mathrm{def}}{=}\log_{2}\left|S_{M}\right| \end{array}$$
  (e.g., for a gas with $N\approx 10^{27}$ particles in a room-sized of volume V, $|S_{M}|\propto V^{N}$, so $H(M)\approx N\log_{2}V\approx 10^{27}$).
  Shannon [2] shifted the idea of entropy to informational uncertainty defining
    (2) $$\begin{array}{c} H(M)\overset{\mathrm{def}}{=}\sum_{\alpha \in S_{M}}\rho (\alpha )\log_{2}\frac{1}{\rho (\alpha )} \end{array}$$
    measuring the expected number of bits of information needed to specify a randomly chosen microstate α given that it is already known that $\alpha \in S_{M}$. The intuition is that if all microstates α had the same probability ρ(α), their number would be $\left|S_{M}\right|=1/\rho (\alpha )$, and the optimal code length for transmitting α would be $\log_{2}\left(1/\rho (\alpha )\right)$ bits.
**Kolmogorov Complexity:** Unlike entropy, which measures the information content of a distribution of objects, Kolmogorov Complexity measures the information content of a single object.
**Micro-Complexity K(*α*):** The Kolmogorov Complexity K(α) of microstate α is defined as the length of the shortest prefix-free program $M_{\alpha }()$ that outputs α. Let U be a fixed universal Turing machine. A program $q_{\alpha }$ is a binary string such that $U(q_{\alpha })=\alpha$. Prefix-Free: No valid program is a prefix of another. This allows programs to be concatenated and unambiguously separated.
  This is assumed to be enormous.
**K(*P*) and K(*M*)** denote the length of the shortest program $M_{P}(\alpha )=p$ that computes property P and both the probability $M_{M}(\alpha ,\epsilon )\approx \rho (\alpha )$ for macrostate M and membership $M_{M}(\alpha )\in \left\{0,1\right\}$ in $S_{M}$.
  For example, a very simple program $M_{\mathrm{temperature}}(\alpha )$ computes the temperature of a gas from the velocity of each of its particles.
**K(*p*)** denote the length of the shortest program $M_{p}()=p$ that outputs the value p. Note that some numbers like Ackermann(5) have short programs relative to their values.
  For example, the number of gas particles $p=10^{27}$ could be represented by the size K(p)≤𝒪(log p) of its binary description or, even better, by K(p)≤𝒪(loglog p)=𝒪(log 27) bits. The latter program, for example, could just output the number 27, specifying $p\approx 10^{27}$.
**Macro-Complexity K(*M*, *P*, *p*)** denotes the length of the shortest program that does all three.
  This is assumed to be small.
**Results Presented:** Though they have very different formulations the entropy H(M) of a macrostate and the Kolmogorov complexity K(α) of its microstates are closely related.
  Levin’s Coding Theorem 1: $K(\alpha )\leq -\log_{2}\rho (\alpha )+K(M)+c$
      Sandwich Theorem 2: $H(M)\leq {\mathrm{Exp}}_{\alpha ∼\rho }K(\alpha )\leq H(M)+K(M)+c=H(M)+c_{M}$
    Uniform Case Theorem 3: ${\mathrm{Max}}_{\alpha }K(\alpha )-c_{M}\leq H(M)\leq {\mathrm{Avg}}_{\alpha }K(\alpha )$
                  ${\mathrm{Var}}_{\alpha }K(\alpha )\leq {c_{M}}^{2}+4c_{M}+6$
  The Not Alone Theorem 4: $2^{K_{\mathrm{Max}}-c_{\langle M,P,p\rangle }}\leq \left|S_{p}\right|\leq 2^{K_{\mathrm{Avg}}}.$
  See Section 4.1, Section 4.2, Section 4.3 and Section 4.4.
**Natural:** In natural physical scenarios, we assume the micro-complexity K(α) is enormous while the total macro-complexity K(M,P,p) is small.
***c* ≈ 1000,** $c_{M}=K\left(M\right)+c$**, and** $c_{\langle M,P,p\rangle }=K\left(M,P,p\right)+c$ are assumed to be “constants”.
**Tight:** This assumption is what makes the Sandwich Theorem $E_{\alpha ∼\rho }K(\alpha )\approx H(M)$, for example, tight.

## 3. Extreme Cases

The following examples illustrate the results in extreme regimes:

**Counting Strings of a Given Complexity,** $\left|\left\{\alpha :K\left(\alpha \right)=k\right\}\right|\leq 2^{k}$**:**
  The number of microstates with complexity k is at most $2^{k}$. This is because each such microstate requires a unique prefix-free program $q_{\alpha }$ of length k and there are at most $2^{k}$ such programs available.
**Random Strings:** An n-bit string α is considered random if it is incompressible, i.e., K(α)≈n. Most strings are random in this sense. The fraction of n-bit strings that can be compressed by more than c bits is at most $2^{-c}$ because there are fewer than $2^{n-c}$ prefix-free programs of length less than n−c.
**Strings with 49% Zeros:** Let $M_{49\%}$ be the macrostate of all n-bit strings containing exactly 49% zeros for some fixed value of n. Then, by Stirling’s approximation,
  |SM49%|=n0.49n⇒H(M49%)=log2|SM49%|≈H(0.49)·n≈0.9997n
  where H(p) is the binary entropy function. Because a short program can check the 49%-zero condition, we know that $K(M_{49\%})$ is small. Given a particular such string α, it is not clear how one would write a short program that outputs it. Levin’s Coding Theorem 1, however, gives such a program of length $K(\alpha )\leq -\log_{2}\rho (\alpha )+K(M)+c=\log_{2}\left|S_{M_{49\%}}\right|+\mathrm{small}\;\mathrm{constant}\approx 0.9997n$. This is only an upper bound, as some strings in $S_{M_{49\%}}$ may be highly compressible (e.g., a string of 0.49n zeros followed by 0.51n ones).
**All Microstates:** Let $M_{n}$ be the macrostate of all microstates α of length n. The number of such strings $|S_{M_{n}}|$ is $2^{n}$, so $H\left(M_{n}\right)=\log_{2}\left|S_{M_{n}}\right|=n$. Most such strings have complexity K(α)=n. The complexity $K(M_{n})$ of the macrostate itself is the size of the program that checks if α has length n. This is at most $c_{M}=K(M_{n})=𝒪\left(\log\;n\right)$, as it only needs to encode the value n. Here, Theorem 1 is tight in the natural extreme.
  $$\begin{array}{c} n-c_{M}\approx K\left(\alpha \right)-K(M_{n})\leq H(M_{n})\approx n \end{array}$$
**Single-Element Macrostate:** Let $M_{\alpha }$ be the macrostate that accepts only one microstate α. Then, $H(M_{\alpha })=\log_{2}\left|S_{M_{\alpha }}\right|=\log_{2}1=0$. Any program to check membership in $M_{\alpha }$ must effectively encode α, so $K(M_{\alpha })\approx K(\alpha )$. In this case, Theorem 1 is tight in the unnatural extreme case. The inequality $K(\alpha )-K(M_{\alpha })\leq H(M_{\alpha })$ becomes
  $$\begin{array}{c} K(\alpha )-K(\alpha )\approx 0\leq 0 \end{array}$$

## 4. Theorems and Proofs

### 4.1. Levin’s Coding Theorem 1

Levin provides a foundational (though confusing) result in algorithmic information theory. It links the complexity of a string α to its *universal probability* m(α)—the probability that a universal Turing machine with random input will output α [6]. Moreover, Chaitin [5] shows that this universal probability m multiplicatively dominates all other computable probabilities like our distribution ρ. Together, the theorem states

$$\begin{array}{c} K(\alpha )\leq -\log_{2}m\left(\alpha \right)+c_{U}\leq -\log_{2}\rho \left(\alpha \right)+c_{\rho }, \end{array}$$

where the constant $c_{U}$ depends only on the choice of the Universal Turing and $c_{\rho }$ also depends on the machine computing the distribution ρ. The key thing is that they do not depend on the string α. Diving into the proof, one can see that $c_{\rho }=K(M)+c$, where K(M) is precisely the length of the program $M_{M}(\alpha ,\epsilon )$ needed to approximate the probabilities ρ(α) and c is maybe 1000. This gives the revised statement:

**Theorem** **1** (Levin’s Coding)**.** *For any distribution ρ and microstate $\alpha \in S_{M}$,*

*we have $K(\alpha )\leq -\log_{2}\rho (\alpha )+K(M)+1003$.*

Qualitatively, this theorem establishes a “conservation of complexity” relative to probability. It states that an object cannot be both simple (low K) and improbable (low ρ) unless the distribution itself is complex. In physical terms, if a microstate is highly probable, it must have a relatively short description.

A quick proof sketch would go as follows: By definition, we give this bound on K(α) by giving a program $M_{\alpha }()$ that outputs α described with $-\log_{2}\rho (\alpha )+K(M)+c$ bits. To $-\log_{2}\rho (\alpha )$ bits of precision, our program is given the cumulative probability $q_{\alpha }=\sum_{\alpha^{\prime }<\alpha }\rho \left(\alpha^{\prime }\right)$. With K(M) bits, it is given the program $M_{M}\left(\alpha ,\epsilon \right)$ needed to approximate the probabilities ρ(α). The remaining 1000 bits describe our program that enumerates through the strings $\alpha^{\prime }$ computing this sum until the target α is reached. What remains is to ensure that all the values are accurate enough so that this works. The more detailed proof is as follows.

**Proof.**  The proof is by construction. We will describe a program that generates α and its length will serve as the required upper bound on K(α).

**1.**  **The Description of *α*:** Our description of *α* consists of two parts which are fed to a fixed universal search program:
  **A Program for the Macrostate *M*:** A program $M_{M}$ that for any given $\alpha^{\prime }$ and a precision parameter ϵ computes its probability $\rho (\alpha^{\prime })$. The length of the shortest such program is by definition K(M).
  **An Identifying Target** $q_{\alpha }$**:** A binary string that uniquely identifies α. This string represents a rational number defined as our “target” which is a multiple of $2^{-n^{\prime }}$. We define this precision $n^{\prime }$ as
    $$n^{\prime }\overset{\mathrm{def}}{=}\left\lceil -\log_{2}\rho \left(\alpha \right)\right\rceil +1$$
    This choice ensures that $\rho \left(\alpha \right)\geq 2\cdot 2^{-n^{\prime }}$. The target $q_{\alpha }$ is provided to the search program as a binary string of length $n^{\prime }+1$ bits.
**2.**  **The Search Algorithm:** The Turing Machine $M_{\mathrm{search}}$ is a fixed universal program (of length, say 1000 bits). It takes the program $M_{M}$ and the identifier $q_{\alpha }$ as input. It then performs the following steps:
  1. It iterates through all microstates $\alpha^{\prime }$ in lexicographical order.
  2. For each $\alpha^{\prime }$, it uses the provided program $M_{M}(\alpha^{\prime },\epsilon (|\alpha^{\prime }|))$ to compute an approximation $\tilde{\rho }\left(\alpha^{\prime }\right)$ of its probability. The required precision $\epsilon (|\alpha^{\prime }|)=\frac{1}{{2|\alpha^{\prime }|}^{2}2^{|\alpha^{\prime }|}}$ ensures that $|\rho \left(\alpha^{\prime }\right)-\tilde{\rho }\left(\alpha^{\prime })\right|\leq \epsilon (|\alpha^{\prime }|)$.
  3. It maintains a running sum of these approximate probabilities.
  4. The algorithm halts and outputs the current microstate $\alpha^{\prime }$ at the exact moment this running sum surpasses the target value represented by $q_{\alpha }$. We claim this procedure outputs our intended microstate α.
**3.**  **Correctness of the Search:** We now prove that this search algorithm correctly and uniquely identifies α.
  **Interval Width:** The interval corresponding to our target microstate α has width $\tilde{\rho }\left(\alpha \right)$. We prove this is at least $2^{-n^{\prime }}$.
    $$\mathrm{Interval}\;\mathrm{Width}=\tilde{\rho }\left(\alpha \right)\geq \rho \left(\alpha \right)-\epsilon \left(|\alpha |\right)\geq 2\cdot 2^{-n^{\prime }}-2^{-|\alpha |}\geq 2^{-n^{\prime }}$$
    We know $n^{\prime }<\left|\alpha \right|$ or else our theorem is trivially true.
  **Defining the Target** $q_{\alpha }$**:** Imagine placing markers on the real number line at every integer multiple of $2^{-n^{\prime }}$. Because the interval for α has a width greater than $2^{-n^{\prime }}$, it is guaranteed to contain at least one such marker. We define $q_{\alpha }$ to be the value of one such marker. When the search algorithm’s running sum surpasses this value, it must have just finished adding $\tilde{\rho }\left(\alpha \right)$ and it correctly outputs α.
  **Bounding |*q_α_*|:** Recall $q_{\alpha }$ is a binary string representing a rational number. The number of bits to the right of the decimal is at most its precision $n^{\prime }=\left\lceil -\log_{2}\rho \left(\alpha \right)\right\rceil +1$. The number of bits on the left is one because the value $q_{\alpha }$ is less than 2, namely $q_{\alpha }\leq \sum_{\alpha^{\prime }}\tilde{\rho }\left(\alpha^{\prime }\right)\leq \sum_{\alpha^{\prime }}(\rho \left(\alpha^{\prime }\right)+\epsilon (|\alpha^{\prime }|))$. The sum of probabilities $\sum_{\alpha^{\prime }}\rho \left(\alpha^{\prime }\right)$ is at most 1. The sum of the errors is bounded: $\sum_{\alpha^{\prime }}\epsilon (|\alpha^{\prime }|)=\sum_{n=1}^{\infty }2^{n}\cdot \frac{1}{2n^{2}2^{n}}=\sum_{n=1}^{\infty }\frac{1}{2n^{2}}=\frac{\pi^{2}}{12}<1$.
**4.**  **Conclusion:** The program $M_{\alpha }$ that produces α is the fixed search program $M_{\mathrm{search}}$ with $M_{M}$ and $q_{\alpha }$ hard-wired in. Hence, $K(\alpha )\leq \;|M_{\alpha }|$ $=\left|M_{\mathrm{search}}\right|+\left|M_{M}\right|+\left|q_{\alpha }\right|$ $\leq 1000+K(M)+\left(n^{\prime }+1\right)$ $=1000+K(M)+\lceil -\log_{2}\rho \left(\alpha \right)\rceil +2$ $<1000+K(M)-\log_{2}\rho \left(\alpha \right)+3$.

□

### 4.2. The Sandwich Theorem 2

We now explain and prove the Sandwich Theorem stating that the expected Kolmogorov complexity K(α) of a randomly chosen microstate α is tightly “sandwiched” by the entropy H(M) of the macrostate.

**Theorem** **2** (Sandwich Theorem)**.** *$H\left(M\right)\leq {Exp}_{\alpha ∼\rho }K(\alpha )\leq H\left(M\right)+K\left(M\right)+c\leq H\left(M\right)+c_{M}$*

*where $c_{M}=K\left(M\right)+1003$.*

In simple terms, this theorem confirms that the “typical” complexity of a microstate matches the entropy of its macrostate. This aligns with the physical intuition that for a gas in equilibrium, the complexity of a snapshot of the system is effectively determined by the volume of the phase space (entropy).

**Proof.**  (Upper Bound) We move from the result at the micro level back to the macro level by taking the weighted sum of Levin’s Coding Theorem 1 with respect to ρ giving

$$\begin{array}{cc} K\left(\alpha \right) & \leq -\log_{2}\rho (\alpha )+K(M)+c\\ {\mathrm{Exp}}_{\alpha ∼\rho }K\left(\alpha \right) & \leq \sum_{\alpha }\rho \left(\alpha \right)\left(-\log_{2}\rho \left(\alpha \right)\right)+K\left(M\right)+c=H\left(M\right)+K\left(M\right)+c \end{array}$$

(Lower Bound) We compare the expected code lengths for two methods of assigning codewords to each microstate $\alpha \in S_{M}$. The first method uses the shortest program $q_{\alpha }$ for each microstate α as its codeword. Recall that we required such programs to be prefix-free. By definition, the expected code length for this method is ${\mathrm{Exp}}_{\alpha ∼\rho }\left[|q_{\alpha }|\right]={\mathrm{Exp}}_{\alpha ∼\rho }K(\alpha )$. The second method is Shannon’s code, which assigns to each microstate α a codeword of the ideal length $-\log_{2}\rho (\alpha )$. By definition, its expected code length is ${\mathrm{Exp}}_{\alpha ∼\rho }\left[-\log_{2}\rho (\alpha )\right]=\sum_{\alpha \in S_{M}}\rho (\alpha )\left(-\log_{2}\rho (\alpha )\right)=H(M)$. Because Shannon’s method provides the optimal expected code length [2], the expected length of the first code must be greater than or equal to the second. □

**Kolmogorov-Based Conditional Entropy**: Rearranging Theorems 1 and 2 gives the difference K(α)−K(M), which rings of conditional entropy. Hence, let us define it to be $H_{K}(\alpha |M)$.

We denoted Shannon’s entropy of a macrostate by H(M) to emphasize it is a function of the macrostate’s distribution. Shannon himself might prefer the *conditional entropy* notation $H_{S}(\alpha |M)$, viewing it as the expected bits needed to specify a randomly chosen α given M. He might express this as the difference between the information needed for α and that for M:

$$\begin{array}{c} H_{S}(\alpha |M)\overset{\mathrm{def}}{=}{\mathrm{Exp}}_{\alpha }\left[\mathrm{BitsToDescribe}(\alpha )\right]-{\mathrm{Exp}}_{\alpha }\left[\mathrm{BitsToDescribe}(M)\right] \end{array}$$

Conditional entropy is normally defined as H(Y|X)=H(X,Y)−H(X). Here, we associate Y with α and X with M. We assume learning α determines M, so H(α,M)≈H(α). Our notation ${\mathrm{Exp}}_{\alpha }\left[\mathrm{BitsToDescribe}\left(\cdot \right)\right]$ is an intuitive stand-in for the standard entropy H(·).

Replacing ${\mathrm{Exp}}_{\alpha }\left[\mathrm{BitsToDescribe}\right]$ with K gives

(3) $$\begin{array}{cc} H_{K}(\alpha |M) & \overset{\mathrm{def}}{=}K(\alpha )-K(M) \end{array}$$

Theorem 2 then becomes

$$H\left(M\right)\approx {\mathrm{Exp}}_{\alpha ∼\rho }H_{K}(\alpha |M)$$

This definition aligns with the standard AIT chain rule. Since the macrostate M is computable from α, K(M|α)≈O(1). The symmetry of information states K(α,M)≈K(M)+K(α|M). Since K(α,M)≈K(α), this yields K(α|M)≈K(α)−K(M), matching our definition.

### 4.3. The Uniform Case Theorem 3

The following are fun improvements when the distribution is uniform: the maximum and average complexities are close and the variance of complexity is tightly bounded by a small constant. This is in stark contrast to the non-uniform case. There exist “natural” macrostates with small K(M) and small average complexity K(α), but infinite variance. For every n, define $\rho_{n}=\frac{0.746}{n^{2.5}}$ and for every n bit string α, define $\rho \left(\alpha \right)=\frac{\rho_{n}}{2^{n}}$. Then, $E_{\alpha ∼\rho }K\left(\alpha \right)\approx \sum_{n=1}^{\infty }\rho_{n}\cdot n=\sum_{n=1}^{\infty }\frac{0.746}{n^{1.5}}\approx 1.95$ and $\mathrm{Var}\left(K(\alpha )\right)=E_{\alpha ∼\rho }{\left(K(\alpha )-1.95\right)}^{2}$$\approx \sum_{n=1}^{\infty }\rho_{n}\cdot n^{2}=\sum_{n=1}^{\infty }\frac{0.746}{n^{0.5}}=\infty$ The first result is also key for our Not Alone Theorem 4.

**Theorem** **3** (Uniform)**.** *Consider a uniform macrostate M.*

*A: ${Max}_{\alpha }K(\alpha )-c_{M}\leq \log_{2}\left|S_{M}\right|=H\left(M\right)\leq {Avg}_{\alpha }K(\alpha )$*

*B: ${Var}_{\alpha }K(\alpha )\leq {c_{M}}^{2}+4c_{M}+6$*

**Proof.**  (A) Levin’s Theorem 1 states $K(\alpha )-c_{M}\leq -\log_{2}\rho (\alpha )$. Replace K(α) with ${\mathrm{Max}}_{\alpha }K(\alpha )$ by applying the theorem to a microstate α of maximum complexity. Because the distribution is uniform, $S_{M}$ must be finite. Because the distribution is uniform, $H(M)=\log_{2}\left|S_{M}\right|=-\log_{2}\rho (\alpha )$. This gives the required ${\mathrm{Max}}_{\alpha }K(\alpha )-c_{M}\leq \log_{2}\left|S_{M}\right|=H(M)$. Theorem 2 states $H(M)\leq {\mathrm{Exp}}_{\alpha ∼\rho }K(\alpha )$, which because uniform is the same as ${\mathrm{Avg}}_{\alpha }K(\alpha )$.

(B) Partition $S_{M}$ based on their complexity relative to $K_{\mathrm{Avg}}\pm c_{M}$. Let $S_{\mathrm{high}}$ be the set of microstates with complexity $K(\alpha )>K_{\mathrm{Avg}}+c_{M}$. Let $S_{\mathrm{mid}}$ be those with $|K(\alpha )-K_{\mathrm{Avg}}|\leq c_{M}$. For each integer d≥0, let $S_{d}$ be those with complexity exactly $K(\alpha )=K_{\mathrm{Avg}}-c_{M}-d$. Let $K_{d}$ denote this complexity value. From Theorem 3.A, we know $K_{\mathrm{Max}}\leq K_{\mathrm{Avg}}+c_{M}$ and hence $S_{\mathrm{high}}$ is empty. The contribution to the variance from the “middle” set is $\frac{1}{|S_{M}|}\sum_{\alpha \in S_{\mathrm{mid}}}{\left(K\left(\alpha \right)-K_{\mathrm{Avg}}\right)}^{2}\leq \frac{|S_{\mathrm{mid}}|}{|S_{M}|}{c_{M}}^{2}$. This leaves $\sum_{d=0}^{\infty }\frac{|S_{d}|}{|S_{M}|}{\left(K_{d}-K_{\mathrm{Avg}}\right)}^{2}=\sum_{d=0}^{\infty }\frac{|S_{d}|}{|S_{M}|}{\left(c_{M}+d\right)}^{2}=\sum_{d=0}^{\infty }\frac{|S_{d}|}{|S_{M}|}\left({c_{M}}^{2}+2c_{M}d+d^{2}\right)$. Note the total coefficient of ${c_{M}}^{2}$ is $\frac{|S_{\mathrm{mid}}|}{|S_{M}|}+\sum_{d=0}^{\infty }\frac{|S_{d}|}{|S_{M}|}$, which is equal to one. We can now consider the $2c_{M}d+d^{2}$ contribution. There are $\left|S_{d}\right|\leq 2^{K_{d}}$ microstates with this complexity and Theorem 3.A gives that $\left|S_{M}\right|\geq 2^{K_{\mathrm{Max}}-c_{M}}\geq 2^{K_{\mathrm{Avg}}-c_{M}}$. Hence, the probability of encountering such a microstate is at most $\frac{|S_{d}|}{|S_{M}|}\leq \frac{2^{K_{\mathrm{Avg}}-c_{M}-d}}{2^{K_{\mathrm{Avg}}-c_{M}}}=2^{-d}$. The remaining variance is then bounded by $\sum_{d=0}^{\infty }2^{-d}\left(2c_{M}d+d^{2}\right)=2c_{M}\left(\sum_{d=0}^{\infty }\frac{d}{2^{d}}\right)+\left(\sum_{d=0}^{\infty }\frac{d^{2}}{2^{d}}\right)=2c_{M}\left(2\right)+6$, giving the result. □

### 4.4. The Not Alone Theorem 4

This section presents Theorem 2.1.3 in the book [4] in this light. We prove that observable low-complexity macrostates cannot contain lone unobservable high-complexity microstates because otherwise any program for M would have to effectively “name” them, forcing K(M)≳K(α). To avoid this, α must be “hidden” in a large crowd of similar microstates all sharing a simple collective pattern, namely

$$\left[\alpha \in S_{M}\right]\Rightarrow \left[S_{M}\;\mathrm{contains}\;\approx 2^{K\left(\alpha \right)-c_{M}}\;\mathrm{similar}\;\mathrm{microstates}\right]$$

By “similar”, we mean sharing the *exact same value* for any given low-complexity property P(α). Namely, the cluster whose size we bound is defined to be $S_{p}=\left\{\alpha^{\prime }\in S_{M}:P\left(\alpha^{\prime }\right)=p\right\}$ where p=P(α).

**Theorem** **4** (Not Alone)**.** *If not empty, $2^{K_{Max}-c_{\langle M,P,p\rangle }}\leq \left|S_{p}\right|\leq 2^{K_{Avg}}$,*

*where $K_{Avg}$ and $K_{Max}$ are the average and max complexities over $S_{p}$,*

*and $c_{\langle M,P,p\rangle }=K\left(M,P,p\right)+1003$.*

This result formalizes the intuition that “complex things cannot exist in isolation.” If a microstate is complex, it is not special; it is just one of many similar states. Structurally, this implies that high-complexity states form large, homogenous clusters (macrostates), while unique, isolated states must be simple enough to be described individually.

**Example:** For example, any microstate α can be clustered with all those of the exactly same length |α|, the same temperature, the same pressure, the same complexity $K_{t}(\alpha )$, and the same probability ρ(α) simply by considering P(α)=〈|α|, temp(α), pressure(α), $K_{t}(\alpha ),$ ρ(α)〉. Theorem 4 proves that this cluster has a size of at least $2^{K\left(\alpha \right)-c_{\langle M,P,p\rangle }}$. Note that this agrees with the first example in Section 3 that proves that the number of microstates with complexity K(α) is at most $2^{K(\alpha )}$ and, hence, this set is a constant fraction of these.

Here, $K_{t}\left(\alpha \right)$ is defined to be the time-bounded version of Kolmogorov Complexity that requires the program that outputs α to halt within a specified time, e.g., Ackermann(|α|) steps. Unlike K(α), the value $K_{t}\left(\alpha \right)$ is can be computed by a short program that simulates all k-bit programs for the specified time limit t. This means that the complexity K(P) of the property is small. And the set of microstates with unbounded time complexity k is a super set of these.

What might be large is the complexity K(p) of outputting the value p=〈|α|, temp(α), pressure(α), $K_{t}\left(\alpha \right),$ ρ(α)〉. The first value |α| can be denoted n. The next three are at most that. Hence, these values can be outputted by a program of size at most K(n)=logn bits. The fifth value ρ(α) for natural macrostates, we will assume, is at least $2^{-{\left|\alpha \right|}^{𝒪(1)}}$. Outputting it exactly would require too many bits. However, if you are happy with a two approximation of the probability, then $\log\log1/\rho \left(\alpha \right)=𝒪\left(\log_{2}n\right)$ bits does the trick. For a non-natural example, consider a macrostate with probability $\rho \left(\alpha \right)=2^{-N_{\alpha }}$, where $N_{\alpha }$ is the integer value of α. To know the probability, a program must essentially know the entire string. Therefore, the complexity of the probability value is K(ρ(α))≈K(α). The Not Alone Theorem bound $\left|S_{p}\right|≳2^{K\left(\alpha_{\mathrm{Max}}\right)-K\left(p\right)}\approx 2^{K\left(\alpha_{\mathrm{Max}}\right)-K\left(\alpha_{\mathrm{Max}}\right)}=1$ correctly predicts that the cluster size is at most 1.

**Empty Example:** In the previous example, we chose some α and then formed the cluster $S_{p}=\left\{\alpha^{\prime }\in S_{M}:P\left(\alpha^{\prime }\right)=P\left(\alpha \right)\right\}$ with similar properties. This construction ensures that the cluster contains at least α itself. If, instead, we define the cluster based on some chosen property p, then the cluster $S_{p}=\left\{\alpha^{\prime }\in S_{M}:P\left(\alpha^{\prime }\right)=p\right\}$ might be empty. In this case, the theorem is not broken because it only applies when $S_{p}$ is not empty. Alternatively, if property p narrows the cluster to one microstate β, then both $K_{\mathrm{Max}}$ and K(p) equal the complexity K(β), hence the lower bound $2^{K_{\mathrm{Max}}-c_{\langle M,P,p\rangle }}=2^{0}=1$ as needed.

**Proof.**  Theorem 3.A applied to macrostate $M_{p}$ that is defined to be uniform over the cluster $S_{p}$ states $K_{\mathrm{Max}}-c_{\langle M,P,p\rangle }\leq \log_{2}\left|S_{p}\right|\leq K_{\mathrm{Avg}}$. Simply exponentiating gives the result. □

As said, this result is the same as Theorem 2.1.3 in the book [4].

**Theorem** **5** (Book Theorem 2.1.3 [4])**.** *Let A⊆N×N be recursively enumerable and let y∈N. Suppose Y={x|〈x,y〉∈A} is finite. Then, for some constant $c_{A}$ depending only on A for all x in Y, we have $K\left(x|y\right)\leq \log_{2}\left(|Y|\right)+c_{A}$.*

In our setting, A={〈α,P(α)〉}, y=p, $Y=S_{p}$, and x=α.

## 5. Macro- vs. Microstates in Physics

This section explores how macro-level concepts in physics are derived from the micro-level properties of particles. With this, we are able to resolve some of the field’s apparent paradoxes. This perspective reveals that the foundational laws of thermodynamics are not arbitrary but are the necessary statistical consequences of simple rules applied to a complex world, all governed by the universal logic of information.

### 5.1. The Tension Between Discrete and Continuous Physics

We acknowledge a fundamental tension in applying algorithmic complexity (defined on discrete binary strings) to classical physics (defined on continuous variables). Our approach relies on discretization—converting continuous phase space into discrete cells. While this is a standard pedagogical tool, it has limitations. For instance, the “shape” of phase space regions (e.g., fractal strange attractors in chaotic systems) affects how entropy scales with precision in ways that simple box-counting may obscure.

Furthermore, we recognize that Quantum Mechanics offers a more rigorous interface between physics and information, where states are vectors in Hilbert space rather than discrete strings. However, our goal here is not to replace the quantum description but to provide a pedagogical bridge using the accessible language of classical statistical mechanics and algorithmic information theory.

### 5.2. The Precision of Phase Space Approximation

In physics, a microstate α specifies the locations and momenta of all n particles. With three position and three momentum real-valued coordinates per particle, α is a point in $R^{6n}$ space. This is called *phase space*. A macrostate M carves out a subset of this space.

To make this discrete, let $S_{M}$ denote the set of infinitesimal cubes of volume dα that α might be in, and let $S_{M}$ denote a set of corresponding unit-volume cubes. A probability distribution is defined using a probability density function ρ(α), where ρ(α)dα is the probability of the microstate being within α’s infinitesimal cube.

Shannon’s entropy, which is the expected number of bits of information needed to specify a randomly chosen microstate α, would be infinite if one had to specify which of the $|S_{M}|$ infinitesimal cubes α is in. However, it is finite if we only need to name which unit-volume cube it is contained in (giving $\log_{2}\left|S_{M}\right|$ for a uniform distribution). We compute the continuous entropy (or differential entropy) H(M) as follows.

Choose a random α according to the distribution ρ(α). The probability of α being in a specific unit cube is approximately ρ(α) (assuming ρ is roughly constant within that cube). If all unit cubes had this same probability density, then the “number” of such cubes would be effectively 1/ρ(α), and the number of bits needed to specify α’s unit cube would be $\log_{2}\left(1/\rho (\alpha )\right)$. This is the code length allocated to microstate α. The expected number of bits needed is the continuous-case integral:

(4) $$H\left(M\right)\overset{\mathrm{def}}{=}\sum_{x\in S_{M}}\mathrm{Pr}\left(x\right)\mathrm{Bits}\left(x\right)=\sum_{x\in S_{M}}\left[\rho (\alpha )d\alpha \right]\log_{2}\frac{1}{\rho (\alpha )}=\int_{\alpha \in S_{M}}\rho \left(\alpha \right)\log_{2}\frac{1}{\rho (\alpha )}d\alpha .$$

### 5.3. Scaling to N-Particle Systems (Algebra of Macrostates)

We apply these theorems to a system with $N\approx 10^{27}$ particles by analyzing them one at a time. If we assume the particles are (mostly) independent, we can model the total macrostate $M_{total}$ as a cross product of N individual macrostates: $M_{total}=M_{1}\times \cdots \times M_{N}$, where $M_{i}$ is the macrostate for the i-th particle. This “Algebra of Macrostates” shows our theorems scale correctly.

**Independent Cross Product:** For $M_{AB}=M_{A}\times M_{B}$, all key quantities (entropy H, complexity K(M), and average/max microstate complexity K(αβ)) are additive (up to a small constant). The Not Alone Theorem’s bound $2^{K_{\mathrm{Max}}-c_{\langle M,P,p\rangle }}$ remains consistent: the cluster size $|S_{p}|$ becomes multiplicative ($\left|S_{p_{A}}\right|\times \left|S_{p_{B}}\right|$), and since the terms in the exponent are all additive, the theorem correctly predicts this product.
**Dependent Cross Product:** We can also define a dependent cross product $S_{Af}=\left\{\alpha f\left(\alpha \right):\alpha \in S_{A}\right\}$, where f is a simple, computable function (e.g., f calculates a particle’s velocity from its position). Here, complexities increase only by the small K(f). Both the cluster size and the theorem’s bound $2^{K_{\mathrm{Max}}-c_{\langle M,P,p\rangle }}$ remain essentially unchanged.
**Example (The “Complex Property” Limit):** This framework also handles the extreme case. Let f(α) be a function that gives α a unique complex property p. For example, f(α) outputs the integer $N_{\alpha }$ (the value of α), and we define our property as $P(\alpha f(\alpha ))=N_{\alpha }$.
  For any microstate, its cluster $S_{p}$ is a singleton ($\left|S_{p}\right|=1$). The Not Alone Theorem correctly predicts this. Since knowing the property $p=N_{\alpha }$ is the same as knowing α itself, the complexity of the property is enormous: K(p)≈K(α). The theorem’s bound becomes
    $$\left|S_{p}\right|\geq 2^{K_{\mathrm{Max}}-c_{\langle M,P,p\rangle }}\approx 2^{K(\alpha )-K(p)}\approx 2^{K(\alpha )-K(\alpha )}=2^{0}=1$$
    This confirms that a microstate can be “alone” in its cluster, but only if the property defining that cluster is just as complex as the microstate itself (and thus not a “simple” macrostate property).
**Scaling of** $c_{\langle M,P,p\rangle }$**:** We can address the scaling of $c_{\langle M,P,p\rangle }$ for standard physical properties. If P represents a global quantity like Total Energy $E_{total}$ in an N-particle system, the value p scales with N. However, the number of bits required to describe this value is only 𝒪(log N). Since the microstate complexity K(α) scales linearly with N (i.e., ≈$10^{27}$ bits), the cost of describing the property (≈$\log\;10^{27}$≈90 bits) is negligible. Thus, even for extensive physical properties, the “constant” $c_{\langle M,P,p\rangle }$ remains small relative to the system size, and the “Not Alone” bound remains non-trivial and physically meaningful.

### 5.4. Newtonian Determinism and Coarse-Graining

The Second Law of Thermodynamics states the entropy of a closed system can only increase over time. Let $M_{0}$ be the macrostate with all gas particles initially in a box of volume $V_{0}$, and let $M_{t}$ be the macrostate at time t, after they have dispersed into a room of volume $V_{t}$. Boltzmann would argue that entropy increases by $n\log_{2}\left(V_{t}/V_{0}\right)$ because the number of microstates $|S_{t}|$ increases.

First, let us be clear that the disorder that is measured by entropy does not arise because there are many particles doing the independent things but because there is uncertainty in what they are doing. Section 5.3 argues that instead of considering a system with $n\approx 10^{27}$ particles, we can analyzing these one at a time. If we assume the particles are (mostly) independent, we can model the total macrostate $M_{total}$ as a cross product of N individual macrostates: $M_{total}=M_{1}\times \cdots \times M_{N}$, where $M_{i}$ is the macrostate for the i-th particle. This “Algebra of Macrostates” shows our theorems scale correctly.

An apparent paradox arises in a deterministic, reversible, closed physical system. Because the laws of physics are reversible, no information is gained or lost. The entropy should not change, in violation of the Second Law.

This is best explained by seeing that the Kolmogorov complexity of the microstate $K\left(\alpha_{t}\right)\approx K\left(\alpha_{0}\right)$ and hence the entropy $H\left(M_{t}\right)\approx H\left(M_{0}\right)$ does not change plus or minus a small constant $c_{t}=K\left(t\right)+K\left(Physics\right)$. This is proved by describing a small program $M_{\alpha_{t}}()=\mathrm{Physics}\left(M_{\alpha_{0}}(),M_{t}()\right)$ that outputs $\alpha_{t}$, where $M_{\alpha_{0}}()$ outputs $\alpha_{0}$, $M_{t}()$ outputs t, and $\alpha_{t}=\mathrm{Physics}\left(\alpha_{0},t\right)$ encodes the laws of physics. Being reversible, $\alpha_{0}={\mathrm{Physics}}^{-1}\left(\alpha_{t},t\right)$ gives the other direction. This requires you both know and can express these laws of physics.

**Figure 1:**
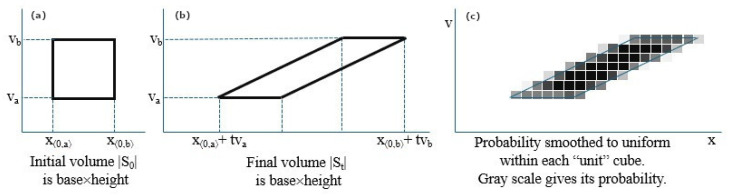
(**a**,**b**) Liouville’s Theorem proves the total volume of the accessible phase space does not change: $\left|S_{t}\right|=\left|S_{0}\right|$. (**c**) Entropy increases through coarse-graining.

Liouville’s fundamental Theorem takes this same $H\left(M_{t}\right)=H\left(M_{0}\right)$ paradox argument a step further. A microstate $\alpha_{t}$ (describing the position and momentum of all n particles) is a point in the continuous $R^{6n}$ phase space. The macrostate $S_{0}$ is the “accessible subregion” of this phase space (e.g., all particles in the box $V_{0}$ with some energy). As time passes, this region evolves to $S_{t}=\mathrm{Physics}\left(S_{0},t\right)$. Even though the particles spread out to fill the larger room $V_{t}$, Liouville’s Theorem proves the total volume of the accessible phase space does not change: $\left|S_{t}\right|=\left|S_{0}\right|$. See Figure 1a,b.

How? Chaos stretches and folds the accessible region $S_{t}$ into a long, skinny, filament-like region that twists and turns throughout the entire phase space, but its total 6n-dimensional volume remains unchanged. Because the differential entropy H(M)=∫ρlog(1/ρ)dα is directly related to this volume, it also does not change. $H\left(M_{t}\right)=H\left(M_{0}\right)$.

The way this paradox is resolved and entropy is seen to increase is through **coarse-graining**. See Figure 1c. Any real measurement has limited precision. We cannot distinguish between points in $S_{t}$ and nearby points that are not in $S_{t}$. We must “blur” our vision. This noise can be modeled as follows:

1. **Thermal Noise:** Boltzmann’s “dust” being knocked around by particle collisions.
2. **Measurement Noise:** Adding Gaussian noise to each value in the final microstate $\alpha_{t}$.
3. **Coarse-Graining:** Making the probability distribution locally uniform within each “measurement cube” (e.g., $1\;{\mathrm{mm}}^{3}$).

If the true accessible phase space $S_{t}$ is a long, skinny filament that twists through the entire room, any of these blurring effects will make it indistinguishable from a uniform distribution over the entire room. This larger, coarse-grained volume is what we perceive as the new, higher-entropy macrostate. This noise also helps restore the assumption of independence between particles, which is lost after they collide.

### 5.5. How Falling Particles Increase Entropy

Let $M_{0}$ denote the macrostate at time 0, representing a gas diffuse in a thin spherical shell of radius $r_{0}$ and area $\Theta ({r_{0}}^{2})$ around the earth. Let $M_{t}$ represent the same gas after the particles have fallen under gravity to a smaller shell of radius $r_{t}$ and area $\Theta ({r_{t}}^{2})$. Being smaller, one might suspect that the volume $|S_{t}|$ of accessible phase space decreases, decreasing entropy, and breaking the Second Law of Thermodynamics that $H\left(M_{t}\right)\geq H\left(M_{0}\right)$.

The paradox seems even worse if we focus on a single particle. Suppose the particle starts with a known velocity of zero, a known height $r_{0}$, and a completely unknown location in the spherical shell. As the particle drops, its height and radial velocity remain known, but its location is only within the shrinking spherical shell. Hence, it takes fewer and fewer bits to communicate the missing location information, implying entropy is decreasing. Knowing such information precisely, however, is *unnatural*. Liouville’s Theorem requires an initial volume $|S_{0}|$, which is zero unless every value has some at least infinitesimal uncertainty.

The resolution comes from *Liouville’s Theorem*, which states that the volume of the 6D phase space (position and momentum) is conserved. It is useful to first consider a simpler case: if gravity were a fixed, parallel acceleration a, it would apply an **additive** change to each microstate’s velocity (v→v+at) and position ($x\to x+\frac{1}{2}{at}^{2}$). This merely shifts or shears the phase space volume $S_{t}$, but does not change its volume. The force of gravity, however, is not a parallel force; it radiates towards the center. This radial force introduces a **multiplicative** scaling, which is resolved by the conservation of *angular momentum*. This is the same principle as a spinning ice skater: when they pull their arms in (decreasing r), they spin faster (increasing angular velocity).

Let us consider the particle’s phase space in polar coordinates. Let $x_{\theta }$ and $x_{\phi }$ denote the location (angle) and $v_{\theta }$ and $v_{\phi }$ denote the angular velocities in the two directions within the spherical shell.

- **Position Space Shrinks:** When the radius r shrinks (e.g., by a factor of 2), the area of the shell shrinks. The range of possible locations, $\Delta x_{\theta }$ and $\Delta x_{\phi }$, also shrinks by this factor.
- **Momentum Space Expands:** By the conservation of angular momentum ($v_{\theta }\cdot r=\mathrm{const}$), as r shrinks by a factor of 2, the angular velocity $v_{\theta }$ must **double** by a multiplicative factor of 2. This causes the range of possible velocity variables, $\Delta v_{\theta }$ and $\Delta v_{\phi }$, to grow by that same factor.

Suppose the initial volume of this 4D slice of phase space is $V_{0}=\Delta x_{\theta }\Delta x_{\phi }\Delta v_{\theta }\Delta v_{\phi }$, then after falling, the new volume is $V_{t}=\left(\Delta x_{\theta }\frac{r_{t}}{r_{0}}\right)\left(\Delta x_{\phi }\frac{r_{t}}{r_{0}}\right)\left(\Delta v_{\theta }\frac{r_{0}}{r_{t}}\right)\left(\Delta v_{\phi }\frac{r_{0}}{r_{t}}\right)=V_{0}$. As promised by Liouville’s Theorem, the multiplicative factors cancel perfectly. The accessible phase space volume $|S_{t}|$ does not change. Hence, the entropy does not either. The Second Law is not violated. The **increase** in entropy, which we observe in reality, only occurs after the fact due to coarse-graining the probability distribution. See Section 5.4.

### 5.6. Momentum, Energy, and Temperature from First Principles

Fundamental physical laws and definitions are linked to statistical realities. We can derive macro-level concepts like momentum, energy, pressure, and temperature from the simple, micro-level properties of particles.

**Temperature vs. Particle Velocities:** A key property of the microstate is the velocity ${\vec{v}}_{i}$ and speed $v_{i}=\left|{\vec{v}}_{i}\right|$ of each particle. The macro-parameter temperature is defined to be the average kinetic energy of these particles, namely $T=\frac{2}{3k_{B}}{\mathrm{Avg}}_{i}\frac{1}{2}m{v_{i}}^{2}$ scaled by the Boltzmann constant $k_{B}=1.38\times 10^{-23}$. When the particles of a gas are at equilibrium, the distribution for $\vec{v}$ is according to the Maxwell–Boltzmann distribution. Formally, this is derived by finding the function that maximizes the system’s entropy (assuming entropy would otherwise increase). A strong physical intuition, however, comes from the Central Limit Theorem. Because it is the result of the “sum” of a vast number of random collisions, each component of the velocity $\vec{v}=\langle v_{x},v_{y},v_{z}\rangle$ is drawn independently from a Gaussian (normal) distribution:
  (5) $$Prob\left(\vec{v}=\langle v_{x},v_{y},v_{z}\rangle \right)\&\propto e^{-\frac{\frac{1}{2}m{v_{x}}^{2}}{k_{B}T}}\times e^{-\frac{\frac{1}{2}m{v_{y}}^{2}}{k_{B}T}}\times e^{-\frac{\frac{1}{2}m{v_{z}}^{2}}{k_{B}T}}=e^{-\frac{\frac{1}{2}m\left({v_{x}}^{2}+{v_{y}}^{2}+{v_{z}}^{2}\right)}{k_{B}T}}\&=e^{-\frac{\frac{1}{2}mv^{2}}{k_{B}T}}=e^{-\frac{E_{k}\left(v\right)}{k_{B}T}}.$$
  A similar factor $\rho \left(x_{r}\right)\propto e^{-\frac{E_{p}\left(x_{r}\right)}{k_{B}T}}$ involving the potential energy of the state is added to the distribution on the height of a particle when there is a force of gravity making it exponentially unlikely for a particle to fly very far up.
**Momentum:** Why is momentum defined as ${\vec{p}}_{i}=m_{i}{\vec{v}}_{i}$? Because this is the quantity that is **conserved** in collisions. When two particles collide, Newton’s third law states they apply equal and opposite forces ($\vec{f}$ and $-\vec{f}$). Since $\vec{f}=m\vec{a}=m\frac{d\vec{v}}{dt}=\frac{d\vec{p}}{dt}$, the two changes in momentum sum to zero. Therefore, the total momentum of the system $\sum_{i}{\vec{p}}_{i}$ remains a constant property of the macrostate.
**Angular Momentum:** Kepler noted that a planet sweeps out equal areas in equal times. This is the conservation of angular momentum. It means that when a spinning ice skater pulls in their arms, they spin faster. Newton (and Richard Feynman) has a fantastic proof involving the area of triangles.
**Energy:** It is reasonable to define potential energy to be $E_{p}=fd$ as it should be linear in the force and distance the object has been pushed. Newton defines kinetic energy to be $E_{k}=\frac{1}{2}{mv}^{2}$ and not $7{mv}^{5}$ because this is the definition that allows for the conservation of energy. For a falling object (constant force f=ma): The change in potential energy is $\Delta E_{p}=-f\cdot \Delta d=-\left(ma\right)\cdot \left(v_{i}t+\frac{1}{2}{at}^{2}\right)$. The change in kinetic energy is $\Delta E_{k}=E_{k}\left(t\right)-E_{k}\left(0\right)=\frac{1}{2}mv{\left(t\right)}^{2}-\frac{1}{2}{mv}_{i}^{2}=\frac{1}{2}m{\left(v_{i}+at\right)}^{2}-\frac{1}{2}{mv}_{i}^{2}=\frac{1}{2}m\left(v_{i}^{2}+2v_{i}at+a^{2}t^{2}\right)-\frac{1}{2}{mv}_{i}^{2}=ma\left(v_{i}t+\frac{1}{2}{at}^{2}\right)$. Thus, $\Delta E_{k}=-\Delta E_{p}$, and the total energy $E_{p}+E_{k}$ is conserved. The total kinetic energy of the system $\sum_{i}\frac{1}{2}m_{i}{v_{i}}^{2}$ is its internal thermal energy.
**Pressure *P*:** Pressure is the force per unit area from particles hitting the container wall. This force is the rate of change of momentum ($\vec{f}=m\frac{\delta \vec{v}}{\delta t}=\frac{\delta \vec{p}}{\delta t}$). Perhaps surprisingly, this depends on ${\mathrm{Avg}}_{i}m{v_{i}}^{2}$ instead of on ${\mathrm{Avg}}_{i}m\left|v_{i}\right|$. The reason is because there are two complimentary effects:
  1. The **frequency** a particle hits a wall is proportional to its speed $v_{i}$ because the ones that are twice as fast reach the wall in half the time, and hence hit the wall with twice the frequency. Consider the sub-volume with area A against the container and infinitesimal height L. If the particles are always uniformly distributed, the expected number in it is $AL\frac{N}{V}$. To avoid worrying about collisions between particles, assume this expected number is much less than one. If there is such a particle moving more or less in the right direction, then the time until collision with the container is $\Theta (\frac{L}{v_{i}})$ and the “rate” of collisions per second is $\Theta (\frac{v_{i}}{L})$.
  2. The **momentum transferred** per hit is also proportional to $v_{i}$. On collision, the perpendicular component of the momentum $\Theta ({mv}_{i})$ is transferred (times two because the particle bounces).

Concluding, the rate of momentum transfer is $\Theta \left(AL\frac{N}{V}\times \frac{v_{i}}{L}\times {mv}_{i}\right)=\Theta \left(A\frac{N}{V}m{v_{i}}^{2}\right)=\Theta \left(A\frac{N}{V}E_{k}\right)$, as needed. This gives that the total force is proportional to $v_{i}\times v_{i}=v_{i}^{2}$. This is convenient because it directly links pressure to the average kinetic energy per unit volume. The exact relation is $P=\frac{2}{3}\frac{N}{V}E_{k}$, where $E_{k}={\mathrm{Avg}}_{i}\frac{1}{2}m{v_{i}}^{2}$ is the average kinetic energy of one particle. Recall $T=\frac{2}{3k_{B}}E_{k}$. This gives the **Ideal Gas Law** $P=\frac{N}{V}k_{B}T$.

## 6. Conclusions

This paper has provided an accessible and computationally grounded framework for understanding the deep connection between entropy and Kolmogorov complexity. Our central contribution is to show that this connection is not merely a mathematical abstraction but a direct consequence of the structural constraints governing how information is described.

Our constructive proof of a tighter Levin’s Coding Theorem reveals the explicit computational cost of specifying a microstate within a macrostate. This naturally leads to the “Not Alone” principle: a simple macrostate cannot contain an isolated complex microstate without its own description becoming complex. Together, these results demonstrate that the statistical properties of a system like its entropy are fundamentally constrained by the algorithmic properties of its individual constituents. They provide a clear intuitive mechanism for why high-complexity states must appear in organized “families” within low-complexity observable systems. We end by exploring concrete properties in physics, resolving a few apparent paradoxes, and revealing how these laws are the statistical consequences of simple rules.

Ultimately, our work reinforces the view that the laws of thermodynamics and information theory are two sides of the same coin both governed by the fundamental rules of computation and description.

### Scope and Limitations

We acknowledge that this framework relies on the standard AIT assumption of a fixed optimal Universal Turing Machine, which introduces additive constants (𝒪(1)) that are negligible only for sufficiently large systems. Furthermore, the mapping of continuous physical systems onto discrete strings requires coarse-graining, the specifics of which can influence the calculated complexity. This work is intended as an interpretive framework to build intuition, rather than a replacement for the rigorous formalisms of statistical mechanics or quantum information theory.

## Data Availability

Data is contained within the article.
